# Fluid management and risk factors for renal dysfunction in patients with severe sepsis and/or septic shock

**DOI:** 10.1186/cc11213

**Published:** 2012-02-29

**Authors:** Laurent Muller, Samir Jaber, Nicolas Molinari, Laurent Favier, Jérôme Larché, Gilles Motte, Sonia Lazarovici, Luc Jacques, Sandrine Alonso, Marc Leone, Jean Michel Constantin, Bernard Allaouchiche, Carey Suehs, Jean-Yves Lefrant

**Affiliations:** 1 Division Anesthésie Réanimation Douleur Urgences, CHU de Nîmes, Place du Professeur Robert Debré, 30029 Nîmes Cedex 9, France. Faculté de Médecine, Université Montpellier 1; 2 Service d'Anesthésie Réanimation B, Hôpital Saint Eloi, CHU Montpellier, 80 avenue Augustin Fliche, 34295 Montpellier, Cedex 5, France; 3 Service de Biostatistique, Epidémiologie clinique, Santé Publique, Information Médicale (BESPIM), CHU de Nîmes, Place du Professeur Robert Debré, 30029 Nîmes Cedex 9, France. Faculté de Médecine, Université Montpellier 1; 4 Service de Réanimation, 2 rue Valentin Haüy, 34 525 Béziers Cedex, France; 5 Service de Réanimation Polyvalente, Centre Hospitalier de Narbonne, 11 Bd du Dr Lacroix, 11100 Narbonne, France; 6 Service de Réanimation Polyvalente, Centre Hospitalier de Perpignan, avenue du Languedoc, 66 000 Perpignan, France; 7 Service de Réanimation Polyvalente, Centre hospitalier Antoine Gayraud, route de Saint-Hilaire, 11890 Carcassonne cedex 9, France; 8 Service de Réanimation, Centre Hospitalier Intercommunal du Bassin de Thau, Bd Camille Blanc, Boite postale 475, 34207 Sète Cedex, France; 9 Service d'Anesthésie et de Réanimation, Hôpital Nord, Aix-Marseille Université, 13915 Marseille cedex 20, France; 10 Service de Réanimation Adultes, CHU Estaing, 1 place Lucie Aubrac, 63003 Clermont Ferrand, France; 11 Service d'Anesthésie Réanimation, Hôpital Edouard Herriot, Hospices Civils de Lyon, 5 place d'Arsonval, 69433 Lyon, France

## Abstract

**Introduction:**

The causative role of new hydroxyethyl starch (HES 130/0.4) in renal dysfunction frequency (a > 50% increase in serum creatinine or need for renal replacement therapy (RRT)) remains debated. Using the database of a multicenter study focusing on patients with severe sepsis and septic shock, the present study aimed at identifying factors associated with the occurrence of renal dysfunction.

**Methods:**

Among the 435 patients in a multicenter study of patients with severe sepsis and septic shock in 15 Southern French ICUs, 388 patients surviving after 24 hour, without a history of renal failure were included. Factors associated with renal dysfunction and RRT were isolated using a multivariate analysis with logistic regression.

**Results:**

Renal dysfunction was reported in 117 (33%) patients. Ninety patients required RRT. Among study participants, 379 (98%) were administered fluids in the first 24 hours of management: HES 130/0.4 only (*n *= 39), crystalloids only (*n *= 63), or both HES 130/0.4 and crystalloids (*n *= 276). RRT was independently associated with the need for vasopressors and the baseline value of serum creatinine in the first 24 hours. Multivariate analysis indicated that male gender, SAPS II score, being a surgical patient, lack of decrease in SOFA score during the first 24 hours, and the interventional period of the study were independently associated with renal dysfunction. Mortality increased in the presence of renal dysfunction (48% versus 24%, *P *< 0.01).

**Conclusions:**

Despite being used in more than 80% of patients with severe sepsis and/or septic shock, the administration of HES 130/0.4 in the first 24 hours of management was not associated with the occurrence of renal dysfunction.

## Introduction

In patients with severe sepsis and septic shock, acute renal failure (ARF) is an independent factor for mortality [1,2]. In the last decade, significant efforts were made to standardize the treatment of septic shock [3,4]. One of the most important recommendations is volume expansion that could also prevent ARF [5,6]. However, the type of fluid, especially the use of colloids, for volume expansion in septic shock remains a matter of debate [7-11]. Indeed, despite a larger plasma volume expansion power [12-14], the use of hydroxyethylstarch (HES) is not related to better outcomes when compared to isotonic crystalloids. In addition, use of HES has been associated with the development of an impaired renal function [7,8,15]. Thus, some experts suggest avoiding the use of HES in septic shock [7-9].

Because recent HES have a lower molecular weight (HES 130/0.4), some authors advocate their use in septic patients [16,17]. In fact, controversial results have been reported in the literature [18,19]. In the field of renal transplant, two studies suggested a better renal tolerance with low molecular weight HES, than with high molecular weight HES or gelatins [20,21]. In patients with septic shock, a recent study suggested that even the use of low molecular weight HES was associated with renal dysfunction. In this study, HES 130/0.4 was used in the first phase of the study when the initial optimization of cardiac preload could be suspected as suboptimal when compared with the final phase using crystalloids [22].

In 2006, a regional program of quality improvement in patients with severe sepsis and septic shock was performed in 15 Southern French ICUs in the Languedoc Roussillon region. After an observational period (six months), an educational program was implemented to optimize the management of patients with severe sepsis and/or septic shock (Sepsi d'Oc study) [23]. As in other studies, the implementation of ten recommendations based on the Surviving Sepsis Campaign [3] and French recommendations were associated with an absolute reduction (13%) of 28-day mortality rates [23].

During this study, information on the type and the volume of fluid was collected in the first 24 hours of patient management. We hypothesized that, in severe sepsis or septic shock patients, the use of HES 130/0.4 may be associated with the development of renal dysfunction. The study was aimed at finding the variables in the first 24 hours that were associated with the occurrence of renal dysfunction in our cohort of patient. The secondary aims were to determine the volume of each type of fluid used in these patients, the relationship between the need for renal replacement therapy (RRT) and the type of fluid, and the outcome and the type of fluid.

## Materials and methods

The present study was a part of Sepsi d'Oc (grants from the University Hospital of Nîmes), a quality improvement program for the management of severe sepsis in 15 ICUs in southern France (Languedoc Roussillon, population: 2,402,000 habitants) [23]. Therefore, the Institutional Review Board at the Nîmes University Hospital approved this study and stated that informed consent was waived. The patients or next of kin were informed of the study and could decline participation in the study.

### The design of the Sepsi d'Oc study

The design of the Sepsis d'Oc study has been described elsewhere [23]. Briefly, the Sepsi d'Oc study compared patients with severe sepsis and/or septic shock who were seen during two periods. During the first six months of 2006 (1 January to 30 June), an observational study was performed while an intervention was proposed in the second half of the year (1 July to 31 December). The intervention was based on the Surviving Sepsis Campaign (Table 1) [3].

**Table 1:** The bundle of 10 recommendations

	Recommendations
# 1	Initial bacteriological samples drawn from sites that are suspected as the source of infection blood cultures in the first three hours
# 2	Initiating an empirical antibiotics therapy in the first three hours
# 3	Measurement of arterial lactate within the first six hours
# 4	Volume expansion ≥20 ml/kg within the first six hours
# 5	A targeted MAP > 65 mmHg within the first six hours
# 6	Assessments of CVP and ScvO2 within the first six hours
# 7	Glucose control ≤8.3 mmol/l within the first 24 hours
# 8	Low doses of corticosteroids when norepinephrine requirement at fewer than six hours
# 9	Using low tidal volume (≤8ml/kg of ideal body weight)
# 10	Adequate use of rhAPC within the first 24 hours

CVP: central venous pressure; MAP: mean arterial pressure; rhAPC: recombinant human activated protein C; ScvO2: oxygen saturation of central venous haemoglobin

### Patients

All patients with severe sepsis and septic shock according to international criteria [3], were eligible for the study. Exclusion criteria were moribund patients, immunosuppression, and evolving severe sepsis or septic shock sepsis for more than 24 hours.

### Measurements

Age, sex, body mass index (BMI), simplified acute physiology II (SAPS II) [24] and sequential organ dysfunction (SOFA) [25] scores, the type (crystalloids or colloids or both) and volume of fluid administered during the first 24 hours of severe sepsis and septic shock were studied. The type of colloids was also collected. The type of fluids used for volume expansion during the first six hours and during the remaining 18 hours was also collected. Patient outcomes were used to determine the 28-day mortality rate and the occurrence of organ failure until day 28 (defined by organ dysfunction and/or infection (ODIN) score [26]).

### The present study (Figure 1)

**Figure 1:**
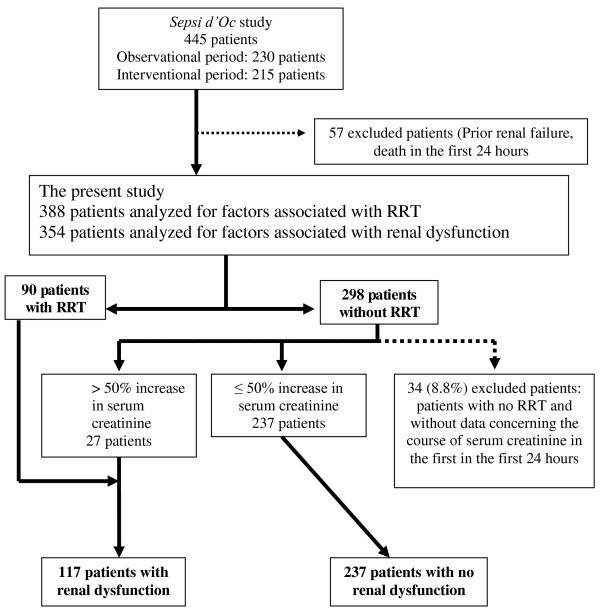
**Flow chart of the study**. RRT, renal replacement therapy.

From the Sepsi d'Oc study data base (institutional free access with anonymized data), we analysed the factors associated with the occurrence of renal dysfunction defined by at least a 50% increase in plasma creatinine concentrations (corresponding to acute kidney injury network (AKIN) stage 1) [27] and/or the need of RRT.

#### Inclusion criteria

Patients included in the Sepsi d'Oc study, without end stage renal disease, who were alive after the first 24 hours of severe and/or septic shock, participated in the present analysis.

#### Exclusion criteria

Patients with end stage renal disease and those who died during the first 24 hours of severe and/or septic shock were excluded.

#### Measured parameters

In addition to variables measured in the Sepsi d'Oc study, the occurrence of renal dysfunction as previously defined was recorded. Information was collected on fluid volumes and the need for vasoactive drugs (vasopressors or inotropes) during the first 24 hours. Moreover, the duration of different organ dysfunctions (ODIN score) until day 28 was also recorded.

### Statistical analysis

Quantitative variables are expressed as means (standard deviation (SD)) or medians (first quartile (Q1), third quartile (Q3)) according to variable distributions. The qualitative variables are expressed as frequencies (percentage).

A univariate analysis was first performed using chi-square tests or Fisher exact tests for qualitative factors and using analysis of variance or Mann-Whitney tests for quantitative factors.

For model building, we applied backward introduction of selected variables from univariate analysis (*P*-entry = 0.20). Data fitting was assessed by the Hosmer Lemeshow test. All analyses were performed using SAS version 9.1 (SAS Institute Inc., Cary, NC) using a 2-sided type 1 error rate of 0.05 as the threshold for statistical significance.

## Results

### Patient population during the study period

In 2006, 6,902 patients were admitted to the 15 ICUs. Five hundred and thirty-eight patients were initially screened for eligibility. Two patients under 18 years of age, 24 patients lacking

one criterion for severe sepsis (infection or Systemic Inflammatory Response Syndrome or organ failure) and 67 patients presenting a non-inclusion criterion were not included. Therefore, the Sepsi d'Oc study involved 445 patients.

### Hemodynamic management

**Figure 2:**
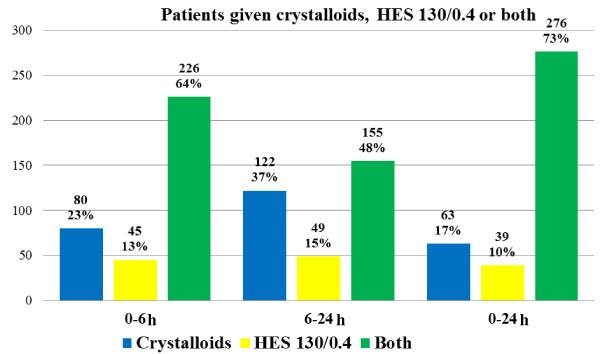
**Number of patients receiving each type of fluid regimen during the initial 24-hour management period**. HES, hydroxyethyl starch.

Among the 445 patients included in the Sepsi d'Oc study, 41 patients had prior end-stage renal disease. Sixteen patients died within the first 24 hours of management. Therefore, 388 patients were included in the present study (202 in the initial observational period, 186 in the second interventional period) (Figure 1). The patient characteristics are shown in Table 2. During the first 24 hours of severe sepsis or septic shock, 379 (98%) out of 388 patients received a fluid administration consisting of exclusively HES 130/0.4 (*n *= 39 (10%)) or crystalloids (*n *= 63 (17%)), or both HES 130/0.4 and crystalloids (*n *= 276 (73%)) (Figure 2). The mean total amount of fluid given during the first 24 hours was 3,780 ± 2,487 ml (Table 3). During the first 24 hours, red blood cell transfusion was required in 90 (24%) patients and vasopressors and inotropes were given to 307 (79%) and 71 (18%) patients, respectively. The 28-day mortality rate was 32%.

**Table 2:** Patient characteristics (data are shown as the median value with interquartile or in absolute value with percentage)

Gender (Male/Female)	257/131	(66/34)
Age (years) (median [interquartile])	66	[54 to 77]
SAPS II at admission (median [interquartile])	44	[37 to 56]
SOFA at admission (median [interquartile])	7	[5 to 9]
ODIN score at admission (median [interquartile])	3	[3 to 4]
Type of admission		
Medical (n, %)	257	66
Elective surgery (n, %)	16	4
Emergency surgery (n, %)	115	30
Site of causative infection		
Pulmonary (n, %)	191	49
Peritonitis (n, %)	76	20
Biliary tract infection (n, %)	13	3
Urinary tract infection (n, %)	39	10
Blood (n, %)	39	10
Central nervous system (n, %)	17	14
Others (n, %)	48	12
Organ failure (with the duration of organ failure)		
Cardiovascular (n, %)	332	86
Duration (median [interquartile])	4	[2-9]
Respiratory (n, %)	336	87
Duration (median [interquartile])	11	[5-21]
Neurologic (n, %)	97	25
Duration (median [interquartile])	6	[3-13]

SAPS II, simplified acute physiology score II; SOFA, sequential organ dysfunction score.

**Table 3:** Volume of fluid administered during the periods 0 to 6 hours and 6 to 24 hours and the total of fluid infused in the first 24 hours.

	0 to 6 hours	6 to 24 hours	Total
HES	938 ± 529	830 ± 731	1,344 ± 951
Crystalloids	1,575 ± 1,355	1,889 ± 1,427	2,965 ± 2,100
Total	2,098 ± 1,597	2,124 ± 1,596	3,780 ± 2,487

The volume of fluid is expressed in milliliters (mean ± standard deviation). HES, hydroxyethyl starch.

### Patients with RRT and renal dysfunction

RRT was required in 90 (23%) out of 388 patients. In the patients who did not undergo RRT, an increase in plasma creatinine of at least 50% was found in 27 (7%) patients. An increase in plasma creatinine of less than 50% was identified in 237 (61%) patients. Of note, the course of the plasma creatinine during the first 24 hours could not be determined in 34 (8.8%) patients (missing data). A renal dysfunction was then confirmed in 117 of 364 patients (32%).

### Factors associated with renal dysfunction

After univariate and multivariate analyses, male gender, an increase in SAPS II scores, surgical patients, no decrease in SOFA scores during the first 24 hours and the interventional period of the study were independently associated with renal dysfunction (Table 4). The mortality rate was higher in patients with renal dysfunction than in those without renal dysfunction (48% versus 24%, *P *< 0.01).

**Table 4:** Univariate and multivariate analyses for renal dysfunction and renal replacement therapy

	Renal dysfunction		Univariate analysis	Multivariate analysis	RRT		Univariate analysis	Multivariate analysis
	**No (*n *= 237)**	**Yes (*n *= 117)**	***P***	**Renal dysfunction** **OR - IC 95%**	**No RRT** ***N *= 298**	**RRT** ***N *= 90**	***P***	**RRT**
Age (mean ± SD)(year)	63 ± 16	67 ± 14	0.03		63 ± 16	68 ± 15	0.03	
**Female (n, %)**	86 (36)	29 (25)		1	108 (36)	23 (25)		
**Male (n, %)**	151 (64)	88 (75)	0.03	2 [1.15 to 3.49]	190 (64)	67 (75)	0.04	
**SAPS II at admission (median with interquartile)**	42 [35-52]	49 [40-61]	< 0.01	1.03 [1.01 to 1.05] per unit	43 [35 to 53]	50 [40 to 63]	< 0.01	
**Type of admission**				1				
**Medical**	171 (72)	67 (57)		3.49 [1.01-12.07]	200 (67)	57 (63)		
**Elective surgery**	6 (3)	7 (6)		1.94 [1.13-3.35]	13 (4)	3 (3)		
**Emergency surgery**	60 (25)	43 (37)	0.01		85 (29)	30 (33)	0.68	
Fluid type (n, %)								
None	5 (2)	2 (1)	0.24		7 (2)	2 (2)	0.89	
Crystalloids	46 (19)	14 (12)			49 (17)	14 (16)		
Colloids	19 (8)	14 (12)			28 (9)	11 (12)		
both	166 (70)	87 (74)			214 (72)	63 (70)		
Use of colloids (n, %)	185 (78)	101 (86)	0.07		241 (81)	74 (82)	0.82	
Transfusion (n, %)								
YES	47(20)	32 (28)	0.07		70 (23)	25 (26)	0.49	
NO	190 (80)	82 (72)			228 (77)	65 (72)		
***Vasopressors (n, %)***								
***YES***	175 (74)	106 (91)	< 0.01		225 (76)	82 (91)	< 0.01	1
***NO***	62 (26)	11 (9)			73 (25)	8 (9)		2.61 [1.18 to 5.77]
Inotropic drug (n, %)								
YES	39 (16)	30 (26)	0.04		49 (16)	22 (24)	0.09	
NO	198 (84)	87 (74)			249 (84)	68 (76)		
**Decrease in SOFA between H_0 _and H_24 _(n, %)**								
**YES**	88 (37)	27 (23)		1	191 (64)	66 (73)		
**NO**	149 (63)	90 (77)	< 0.01	2.49 [1.40-4.41]	107 (36)	24 (27)	0.11	
Baseline mean arterial pressure (mean ± SD) (mm Hg)	69 ± 22	69 ± 20	0.52		69 ± 22	69 ± 21	0.57	
Baseline central venous pressure (mean ± SD) (mm Hg)(MD = 264)	12 ± 5	12 ± 5	0.78		12 ± 5	12 ± 5	0.99	
Baseline arterial lactate (mean ± SD) (mMol/l)(MD = 162)	3.4 ± 2.7	3.6 ± 2.8	0.68		3.4 ± 2.7	3.6 ± 3.0	0.99	
History of renal dysfunction (n, %)								
YES	11 (5)	3 (3)	0.40		12 (4)	3 (3)	1.00	
NO	226 (95)	114 (97)			86 (96)	87 (97)		
***Baseline creatinine (mean ± SD) (mMol/l) ***	122 ± 71	148 ± 84	< 0.01		119 ± 69	159 ± 87	< 0.01	1.006 [1.003-1.009] per unit
Baseline renal SOFA (n, %)			< 0.01				< 0.01	
0 to 1	174 (73)	69 (59)			218 (73)	51 (57)		
2-3 to 4	63 (27)	48 (41)			79 (27)	39 (43)		
**Period of study**								
**Observational**	130 (55)	52 (44)	0.06	1	158 (53)	44 (49)	0.49	
**Interventional**	107 (45)	65 (56)		1.90 [1.14-3.18]	140 (47)	46 (51)		

In bold, factors associated with renal dysfunction, in bold and italic: factors associated with RRT. MD, missing data; OR, odds ratio; RRT, renal replacement therapy; SAPS II, simplified acute physiology score II; SOFA, sequential organ dysfunction score.

### Factors associated with RRT

After multivariate analysis, the need for vasopressors and the baseline value of plasma creatinine were independently associated with the need for RRT (Table 4). The mortality rates were 52% in patients requiring RRT and 26% in those not requiring RRT (*P *< 0.01).

## Discussion

The present study focuses on the factors associated with the occurrence of renal dysfunction in patients with severe sepsis and septic shock. In our cohort, 73% of patients were given a combination of HES and crystalloids. With respect to renal dysfunction [27], male gender, a high SAPS II score, no decrease in SOFA scores, the case-mix (surgery), and the interventional period of the Sepsi d'Oc study were identified as risk factors for renal dysfunction. The factors associated with the need for RRT were the baseline value of plasma creatinine and the need for vasopressors. The administration of HES 130/0.4 in the first 24 hours of resuscitation was not associated with a risk of renal dysfunction.

The Sepsi d'Oc study [23] was aimed at improving the initial management (during the first 24 hours) of patients with recent severe sepsis and/or septic shock (< 24 hours). Based on the Surviving Sepsis Campaign [3], ten recommendations were implemented in 15 ICUs of the Southern French 'Languedoc Roussillon' region. This before-after study resulted in a 28 day-mortality reduction. This finding was concordant with those of recent, large studies [28-32].

In the first 24 hours of management, more than 80% of the patients received HES. The volume of colloids was 830 ± 731 ml with a third quartile at 1,800 ml. The use of comparable volumes of HES in patients with severe sepsis or septic shock has already been reported elsewhere [19,33]. In the present study, the volume of infused HES was in the range of recommended doses, as only 2% of patients received more than 50 ml/Kg of HES. Our findings are globally consistent with the available literature, suggesting that our population is well-representative of severe sepsis and septic shock patients. Of note, the total volume during the initial period of severe sepsis and or septic shock was lower than that reported by other groups [29,34]. The occurrence of renal dysfunction and the need for RRT are associated with poorer outcomes, as previously reported [1,2,35].

In the Sepsi d'Oc study, the interventional period was associated with a larger occurrence of renal dysfunction but a higher 28-day survival rate. This finding suggests that an optimization of the initial management of patients with severe sepsis and septic shock may decrease the impact of renal dysfunction on the outcomes, as recently suggested by Badin *et al.*[36]. Moreover, the decrease in the 28-day mortality rate could expose more patients to the risk of renal dysfunction.

The present study failed to show that a low molecular weight HES is associated with poor renal outcomes. Initially, the potential deleterious effect of HES was demonstrated in the renal graft setting with previous non-low molecular weight types of HES [37,38]. The suggested mechanism was an osmotic nephrosis. In ICU patients with septic shock, Schortgen *et al.*[39] also reported that more renal dysfunction was predominant in patients receiving HES, but this did not result in an increase in mortality or the need for RRT. In the VISEP study [15], a higher rate of renal dysfunction was reported but this did not impact the 90-day mortality rate. These previous studies used high molecular weight HES that were sometimes given in extremely large doses.

Some studies have shown that HES 130/0.4 is not associated with the development of impaired renal function [18,19]. In the renal graft setting, two studies have shown that HES 130/0.4 is less deleterious in terms of renal function than the old generations of HES and gelatins [20,21]. Elsewhere, in ICU patients, three studies reported that HES 130/0.4 administration was associated with poor renal outcome [8,22,40]. In the study reported by Bayer *et al.*[22], the baseline Central Venous Pressure (CVP) value was lower and more albumin and platelets and fresh frozen plasma were administrated in the HES group than in the gelatin and crystalloids groups. Moreover, patients in the HES group were more exposed to nephrotoxic product. These differences could explain why more renal dysfunctions were observed in this group without an increase in the need for RRT. In the study reported by Shortgen *et al.*[40], patients requiring HES administration previously received larger amounts of fluids. Moreover, they required more mechanical ventilation suggesting that they were more seriously ill whereas the SAPS II score was similar. In contrast, other groups reported similar findings to those of the present study [18,19]. In the SOAP study [41], the infusion of HES was more frequent in the most seriously ill patients (higher baseline SAPS II and SOFA scores, more mechanically ventilated patients, more patients requiring blood products, more patients with severe sepsis and more patients with shock during their ICU stay) [18]. However, the multivariate analysis did not find that HES were associated with an increased risk of renal dysfunction. The study reported by Boussekey *et al.*[19] also failed to find a deleterious effect of HES in patients with severe sepsis and/or septic shock. Moreover, volumes of infused HES were similar to those reported in the present study (763 ± 595 ml on day 2, 1,031 ± 800 ml on day 7, 1,361 ± 1,393 ml on day 21). Our study suggests that in a real life situation, the physicians tend to respect the recommended doses of HES. This could probably partly explain the different results with previous studies in which HES was widely given. The present study clearly shows that the use of adequate dosages of HES is not associated with the occurrence of renal dysfunction.

The present study has several limitations. First, it focused on the use of different therapies in the first 24 hours of initial management of patients with severe sepsis and/or septic shock. We cannot report the real volume of fluid after 24 hours. However, Boussekey *et al.*[19] showed that the main dosage of HES is administrated in the first two days. Second, the present study was a cohort study and several biases could interfere with the findings. The physicians were not blinded, and a pre-selection of patients may have played a role in our results. However, this is a study reporting real-life practice, giving a partial response to an unresolved issue. One should note that more than 70% of our patients received both colloids and crystalloids, while randomized clinical trials often favor the use of a single type of fluid [15,39]. Even if a multivariate analysis was performed, further randomized clinical trials will probably provide a definitive answer.

## Conclusions

In the present study, renal dysfunction was independently associated with male gender, SAPS II score, a surgical patient, no decrease in SOFA score in the first 24 hours, and the interventional period of the Sepsi d'Oc study whereas RRT was independently associated with the need for vasopressors and the baseline value of serum creatinine. HES was widely used in patients with severe sepsis and septic shock in the initial 24-hour period but was not associated with deleterious impacts on renal function.

## Key messages

• During the first 24 hours of severe sepsis or septic shock, 379 (98%) of 388 patients received fluid administration consisting exclusively of HES 130/0.4 (*n *= 39 (10%)) or crystalloids (*n *= 63 (17%)), or both HES 130/0.4 and crystalloids (*n *= 276 (73%)). The mean total amount of fluid given during the first 24 hours was 3,780 ± 2,487 ml. The overall mortality rate was 32%.

• RRT was required in 90 (23%) of 388 patients. The need for vasopressors and the baseline value of plasma creatinine were independently associated with the need for RRT. The mortality rates were 52% in patients requiring RRT and 26% in those not requiring RRT (*P *< 0.01).

• A renal dysfunction was diagnosed in 117 of 364 patients (32%) (34 patients were excluded because of missing information concerning the initial course of plasma creatinine). After multivariate analysis, male gender, an increase in SAPS II scores, surgical patients, no decrease in SOFA scores during the first 24 hours and the interventional period of the Sepsi d'Oc study were independently associated with renal dysfunction. The mortality rate was higher in patients with renal dysfunction than in those without renal dysfunction (48% versus 24%, *P *< 0.01).

• The use of HES was not associated with RRT or renal dysfunction.

## Abbreviations

AKIN: acute kidney injury network; ARF: acute renal failure; BMI: body mass index; HES: hydroxyethyl starch; ODIN: organ dysfunction and/or infection; RRT: renal replacement therapy; SAPS II: simplified acute physiology score II; SOFA: sequential organ dysfunction score

## Competing interests

The authors declare that they have no competing interests.

## Authors' contributions

LM, SJ, NM and JYL designed the study. LM, SJ, SA, NM and JYL participated in the statistical analysis. LM, SJ, NM, ML, CS and JYL wrote the manuscript. ML, BA, JMC helped to review the final version of the manuscript. All authors participated in the enrollment of patients and in the acquisition of data. All authors have read and approved the final manuscript.
